# Muscle Architecture Properties of the Deep Region of the Supraspinatus: A Cadaveric Study

**DOI:** 10.1177/23259671241275522

**Published:** 2024-10-14

**Authors:** Isabella T. Wu, Sydnee A. Hyman, Mackenzie B. Norman, Gabriela Sendek, Jenna J. Powell, Tyler N. Kirchberg, David B. Berry, John G. Lane, Anshuman Singh, Samuel R. Ward

**Affiliations:** * Department of Orthopaedic Surgery, University of California, San Diego, San Diego, California, USA; † Department of Bioengineering, University of California, San Diego, San Diego, California, USA; ‡ Department of Nanoengineering, University of California, San Diego, San Diego, California, USA; § Department of Orthopaedic Surgery, Kaiser Permanente, San Diego, San Diego, California, USA; || Department of Radiology, University of California, San Diego, San Diego, California, USA; Investigation performed at the University of California, San Diego, San Diego, California, USA

**Keywords:** rotator cuff, muscle architecture, sarcomere length, supraspinatus, anatomy

## Abstract

**Background:**

The supraspinatus is most frequently involved in rotator cuff tears, a common orthopaedic condition. However, the architecture of this muscle has been described only for the superficial, anterior, and posterior regions.

**Purpose:**

To determine the muscle architecture of the deep supraspinatus.

**Study Design:**

Descriptive laboratory study.

**Methods:**

Muscle architecture measurements were collected from 25 cadaveric supraspinatus specimens (13 intact [without tendon tears], 3 with partial-thickness tears, 9 with full-thickness tears). The muscle was divided into deep, superficial anterior, and superficial posterior regions. Pennation angle, raw and normalized fiber length, and sarcomere length and number were compared using repeated-measures analyses of variance.

**Results:**

First, mean architecture measurements were compared between regions using only the intact specimens (n = 13). The deep region had a lower mean pennation angle (3.3° ± 1.0°) compared with the posterior region (11.0° ± 3.9°; *P* < .0001), which in turn had a significantly higher pennation angle compared with the anterior region (7.6 ± 2.6°; *P* = .0005). Normalized fiber lengths in the deep region were 21.1% (*P* = .0052) and 34.5% (*P* < .0001) shorter than the posterior and anterior normalized fiber lengths, respectively. Sarcomere lengths in the deep region were longer (3.4 ± 0.2 μm) compared with the posterior (3.1 ± 0.2 μm; *P* = .0012) and anterior (3.2 ± 0.2 μm; *P* = .0390) regions. Sarcomere numbers also decreased in the deep region by 21.2% (*P* = .0056) and 34.2% (*P* < .0001) compared with the posterior and anterior regions, respectively.

**Conclusion:**

The deep supraspinatus had significantly lower pennation angles, shorter fiber lengths, and fewer but longer sarcomeres in series compared with other subregions within the muscle. These structural differences suggest a functionally unique “submuscle” within the supraspinatus.

**Clinical Relevance:**

Understanding the architecture of the supraspinatus muscle can provide insight into muscle function in health and disease. Specifically, this deep submuscle may play a different role in rotator cuff function than the rest of the muscle.

Rotator cuff tears are common in the older population, and the supraspinatus is the most frequently affected. Understanding the architecture of the supraspinatus muscle can provide insight into muscle function in health and disease. Muscle architecture is defined as the arrangement of muscle fibers relative to the axis of force generation,^14^ with key parameters including mass, muscle fiber length, pennation angle, sarcomere length, and sarcomere number.^7^ The number, length, and arrangement of muscle fibers can accurately predict force generation, excursion, and contraction velocity in the muscle and are required for predicting joint biomechanics.^26^

Muscle atrophy after rotator cuff tears has been widely described. After a tear, muscle fiber length decreases proportionally to tear size.^7^ In full-thickness tears with size <5 cm, this occurs via serial sarcomere subtraction: sarcomere number decreases, but sarcomere length is preserved.^7^ However, in massive tears (>5 cm), sarcomere length additionally decreases.^7^ This further alters the force-length relationship of the muscle relative to joint position. Overall, these changes are expected to further impair muscle force production beyond the fatty degenerative changes commonly identified in the rotator cuff. Moreover, muscle fiber length does not recover, even with successful long-term repair.^7^ Inability to restore the muscle architecture may be a contributor to repair failure in massive tears.^7^ Thus, muscle architecture has important clinical consequences and applications.

With the overall architecture of the supraspinatus muscle becoming better understood, attention now turns to its anatomically distinct regions. Most architecture studies have focused on the anterior versus posterior divisions of the supraspinatus.^10,12,18,20,22,25^ Differences between the superficial and deep areas of the muscle are less well studied. One group of investigators found that “a clear cleavage plane separated the [posterior superior] from the underlying posterior middle part.”^11^ The “posterior superior” part had a significantly different pennation angle from the “posterior middle” and “posterior deep” parts.^11^ The posterior deep part also showed the shortest fiber bundle lengths. However, other key architectural parameters such as sarcomere length and number were not analyzed. Quantifying the architecture of the deep supraspinatus can provide insight into the function of this region.

From an imaging perspective, the deep region can be visualized on high-quality magnetic resonance imaging (MRI) scans, originating from the distal pole of the suprascapular fossa and glenoid neck and inserting onto the deep surface of the tendon (Figure 1). However, this illustration is limited to 2 dimensions and by factors such as slice thickness. We propose that the deep supraspinatus may be better seen on diffusion tensor imaging (DTI) after using a 3-dimensional (3D) postprocessing technique known as *muscle tractography*.^1,24^ Tractography can facilitate better 3D visualization and quantification of different muscle areas. These tools have never been used to identify subcompartments of the rotator cuff muscles.

**Figure 1.**
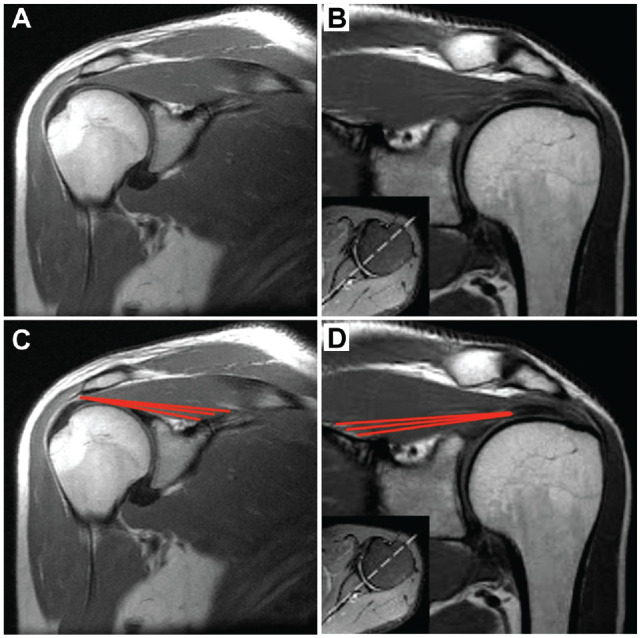
T1-weighted coronal magnetic resonance imaging (MRI) of the supraspinatus muscle. (A, B) Coronal oblique T1-weighted MRI section through the midbelly of the supraspinatus in 2 different patients. (C, D) Red lines indicate the approximate origin and insertion of the deep supraspinatus muscle fibers.

The purpose of this cadaveric study was to determine the architectural properties of the deep and superficial regions of the intact supraspinatus muscle. The hypothesis was that the deep region represents an architecturally distinct—and therefore possibly functionally distinct—component of the muscle. We also investigated the effect of rotator cuff tears on these architectural properties.

## Methods

### Cadaveric Specimens

This study included 25 formalin-embalmed human cadaveric shoulder specimens with a mean age of 87 ± 11 years. There were 10 male and 14 female cadavers, with 1 cadaver’s sex not documented. A total of 15 right and 10 left shoulders were dissected and analyzed.

### Dissection

Superficial muscles (trapezius and deltoid) were removed to expose the rotator cuff. Blunt dissection was used to separate fascial layers, ensuring no damage to the supraspinatus. A bone saw was used to remove the acromion at the scapular notch and fully expose the supraspinatus. With the supraspinatus intact, the following findings were recorded: anterior to posterior dimension of the humeral head, musculotendinous junction length, presence of a tear (full thickness, partial thickness, or none), tear dimensions, and presence or lack of surgical implants in the tear. Then, the supraspinatus was dissected from the bone starting from the vertebral border and superior border of the scapula and moving laterally.

**Figure 2.**
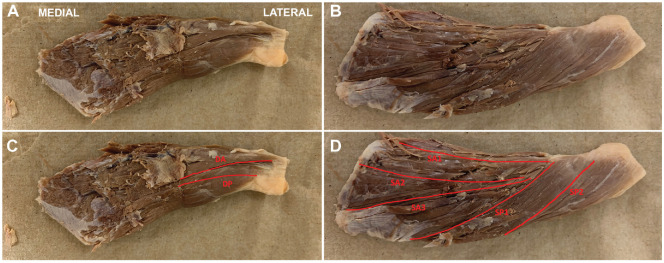
Representative photographs of an isolated supraspinatus muscle after removal of sample fascicles on the (A) superficial and (B) deep surfaces. (C, D) The locations of the 7 individual removed fascicles are identified by red lines, which also represent the fascicle length measured. DA, deep anterior; DP, deep posterior; SA, superficial anterior; SP, superficial posterior.

After the supraspinatus was removed from the fossa, the muscle length, mass, and pennation angle were measured and recorded. Muscle length was measured from the origin of the most proximal fibers to the insertion of the most distal fibers in the tendon. Pennation angle measurement was performed similarly to previous studies.^19,26^ Before fascicles were removed, a goniometer was placed along the length of the fiber and along the force-transmitting axis of the muscle (distal tendon), with the vertex at the point of fascicle insertion to the tendon. Fiber bundles (fascicles) were identified and carefully dissected from the proximal attachment site to the distal tendon insertion. A single fascicle was removed from each superficial or deep region: superficial anterior (SA; with 3 subregions: SA1, SA2, and SA3), superficial posterior (SP; with 2 subregions: SP1 and SP2), deep anterior (DA), and deep posterior (DP) (Figure 2). The superficial regions have been defined in previous studies.^18,22,23^ The deep regions were distinct bundles where the origin was from the lateral-most margin of the supraspinous fossa just medial to the glenoid neck and the insertion was into the distal tendon, as opposed to a separate leaflet of the muscle (Figure 2). After representative fascicles were removed, each was measured using a digital caliper to obtain the raw fiber bundle length (referred to as “raw fiber length”). Whole muscle specimens were stored in 1× phosphate-buffered saline. Individual fascicles were labeled and stored in 1× phosphate-buffered saline at room temperature ahead of sarcomere measurement.

### Normalization

Raw fiber length was normalized to sarcomere length to eliminate joint angle–dependent variations in fiber length.^6^ Previous work has demonstrated that sarcomere length normalization permits resolution of fiber length differences of 15%.^6^ These measurements were made using laser diffraction as previously described.^13,16,26^ Briefly, because of the organization and regularity of the protein lattice structure in muscle, it acts like a diffraction grating. This means that coherent light (ie, laser light) scatters at predictable angles relative to the underlying sarcomere lengths. Measuring the diffraction angle allows the calculation of sarcomere length with high precision. The mean raw sarcomere length of each fascicle was used to normalize the fiber length (*L_f_*) using the following equation^6,26,27^:

$$L_{f}\;=\;L_{f}\prime \;\times \;\left(\frac{2.7\;\mu m}{L_{s}}\right)$$

where $L_{f}\;=\;L_{f}\prime \;\times \;\left(\frac{2.7\;\mu m}{L_{s}}\right)$ is the raw fiber length and *L*_s_ is the measured sarcomere length. An optimal sarcomere length (length at which maximal isometric force production is produced) of 2.7 μm was used for normalization.^26^ Additionally, the sarcomere number was calculated for each region by dividing the fiber length by sarcomere length and multiplying by 1000 for the conversion from millimeters to micrometers.

### Tractography

Diffusion tensor imaging is a quantitative MRI technique that measures the restricted diffusion of water in anisotropic tissues such as muscle. Water molecules within these fascicles/fibers statistically preferentially diffuse along the main axis of a muscle fiber. This technique can be used to quantify and orient (vectorize) the predominant diffusion directionality of muscle fascicles/fibers on a slice-by-slice basis. Tractography is the process of reconstructing the slice-by-slice depictions of these vectors to representative streamlines that can be used to noninvasively visualize and measure muscle fascicles/fibers in 3D.

In this study, a shoulder MRI was performed in a single healthy asymptomatic male volunteer (age, 23 years) using a 3.0-T scanner (Siemens Prisma) with a shoulder coil. A coronal oblique T1-weighted MRI was utilized to visualize the entire rotator cuff (echo time/repetition time = 46 ms/5460 ms, voxel size = 2 × 2 × 4 mm, *b* = 500 s/mm^2^, number of directions = 60). The restricted diffusion profile was calculated using the Analysis of Functional NeuroImages command “3dDWItoDT,” from which tractography was employed to approximate streamlines informed by muscle architecture, as reported previously.^1^ The supraspinatus tractography model was generated in Diffusion Toolkit and visualized in Trackvis.^24^ An angular threshold of 15° was used as termination criteria, and an interpolated streamline propagation algorithm was used.

### Statistical Analysis

Descriptive statistics were calculated and reported for age and gross anatomic measurements. Repeated-measures analysis of variance (ANOVA) was used to compare pennation angle, raw fiber length, sarcomere length, sarcomere number, and normalized fiber length. Mixed-effects analysis was substituted for ANOVA if any data cells were missing. Sidak multiple comparison tests were used for post hoc testing. Analysis was first conducted using all 7 region measurements. As results indicated there were no significant differences between certain regions, the DA and DP were grouped into a “deep” region, SP1 and SP2 into “superficial posterior,” and SA1, SA2, and SA3 into “superficial anterior.” Statistical analyses were performed in GraphPad Prism (Version 9).

## Results

The 25 shoulder specimens had a mean anterior-to-posterior humeral head width of 51 ± 5 mm with a mean musculotendinous junction length of 34 ± 13 mm. The mean supraspinatus muscle length was 121 ± 18 mm, with a mean mass of 29.7 ± 9.0 g. There were 13 specimens with an intact rotator cuff, 9 specimens with a full-thickness tear, and 3 specimens with a partial-thickness tear. The mean tear width was 25 ± 12 mm, and the mean tear length was 29 ± 15 mm. One specimen with a full tear had retained tuberosity implants.

**Appendix Figure A1.**
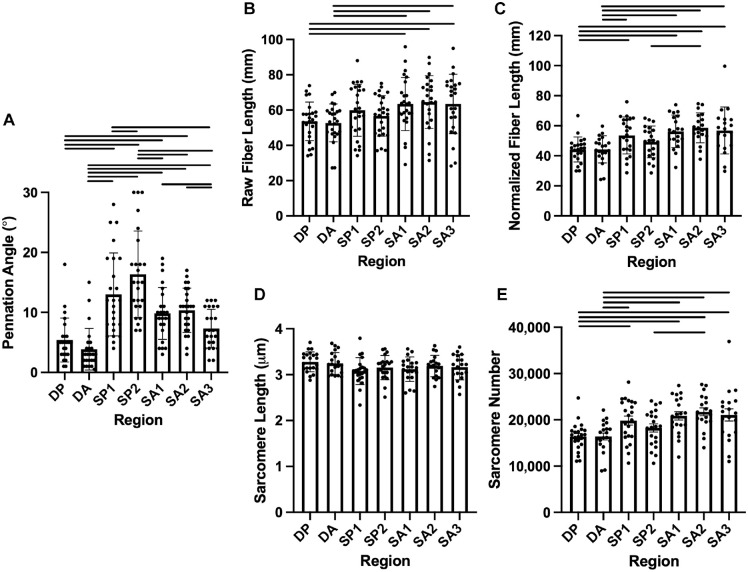
Architecture measurements for all 25 cadavers using the individual region distinctions in pennation (A), raw fiber length (B), normalized fiber length (C), sarcomere length (D), and sarcomere number (E). SA, superficial anterior; SP, superficial posterior; DA, deep anterior; DP, deep posterior. Mean ± standard deviation and significant comparisons (p <0.05) are shown.

The muscle architecture parameters (pennation angle, raw fiber length, sarcomere length, normalized fiber length, and sarcomere number) were compared across all 7 regions (Appendix Figure A1). There were no significant differences on any parameter within each region (eg, DA vs DP, SP1 vs SP2, SA1 vs SA2 vs SA3), except for a larger pennation angle in SP1 compared with SP2 (13.0° vs 16.4°, respectively; *P* = .0418) and SA2 compared with SA3 (10.4° vs 7.3° respectively; *P* = .0093). Because the characteristics were largely similar, these regions were averaged into 3 major regions (deep, posterior, and anterior) for further analysis. Analysis for each architecture parameter was first performed using the 13 intact specimens, with the remaining 12 specimens with tears included in separate subsequent analyses.

### Intact-Only Architecture

**Figure 3.**
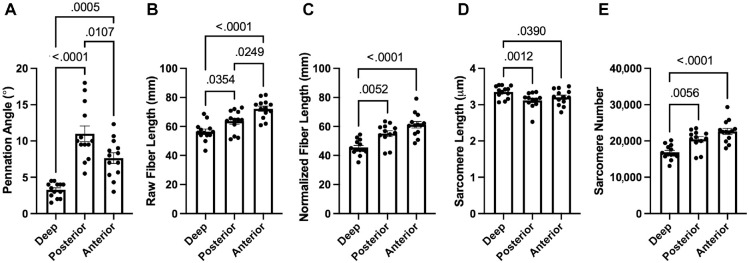
Muscle architecture parameters for the intact-only supraspinatus specimens showed that the deep region had significantly (A) decreased pennation angle, (B) shortened raw and (C) normalized fiber length, (D) longer sarcomere length, and (E) fewer sarcomeres compared with the anterior and posterior regions. Bars represent means and error bars represent SDs. Statistically significant comparisons (*P* < .05) are indicated.

In the specimens with intact rotator cuffs, the deep region showed significant differences compared with the posterior and anterior regions for all architecture parameters (Figure 3). The mean pennation angle was lower in the deep region compared with the superficial posterior region (3.3° ± 1.0° vs 11.0° ± 3.9°; *P* < .0001) and the superficial anterior region (3.3° ± 1.0° vs 7.6° ± 2.6°; *P* = .0005). The raw fiber lengths were shorter in the deep region by 12.6%, compared with posterior (*P* = .0354) and 27.9% compared with anterior (*P* < .0001). Normalized fiber lengths in the deep region were 21.1% shorter than the posterior normalized fiber lengths (*P* = .0052) and 34.5% shorter than the anterior normalized fiber lengths (*P* < .0001). In terms of sarcomere organization, the deep region demonstrated significantly longer and fewer sarcomeres in series. Sarcomeres were shorter in the deep region compared with posterior (3.4 ± 0.2 μm vs 3.1 ± 0.2 μm; *P* = .0012) and compared with anterior (3.4 ± 0.2 μm vs 3.2 ± 0.2 μm; *P* = .0390). Sarcomere number was also decreased in the deep region by 21.2% compared with posterior (*P* = .0056) and 34.2% compared with anterior (*P* < .0001).

### Full- or Partial-Thickness Rotator Cuff Tears

**Figure 4.**
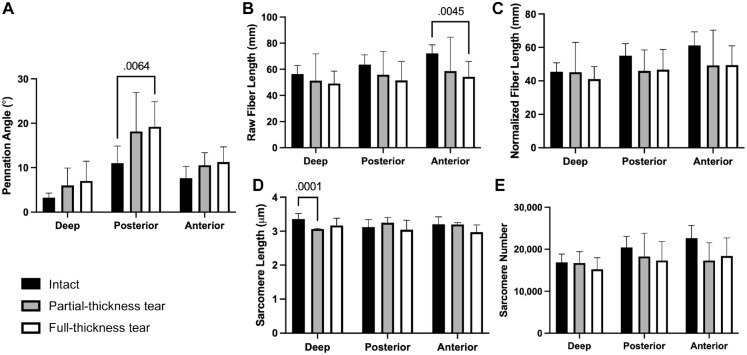
Changes in supraspinatus architecture by region, after a partial- or full-thickness tear compared with intact rotator cuff specimens. (A) Pennation angle in the posterior region was significantly increased after a full tear. (B) Raw fiber length shortened in the anterior region after a full tear, though (C) no difference was observed after normalization. (D) Sarcomere length decreased in the deep region after a partial-thickness tear. (E) No changes in sarcomere number were observed in any region after a tear. Bars represent means and error bars represent SDs. Statistically significant comparisons (*P* < .05) are indicated.

Regional differences were compared between specimens with an intact rotator cuff, full-thickness tear, and partial-thickness tear (Figure 4). A full-thickness tear significantly increased the pennation angle of the posterior region compared with the intact specimen (19.2 ± 14.6° vs 11.0 ± 7.4°; *P* = .0064). Full-thickness tears also resulted in significantly shorter raw fiber lengths in the anterior region compared with intact (54 ± 12 mm vs 72 ± 7 mm; *P* = .0045). However, there were no significant differences in normalized fiber length or sarcomere numbers based on tear status.

### MRI Visualization of an Intact Deep Supraspinatus

**Figure 5.**
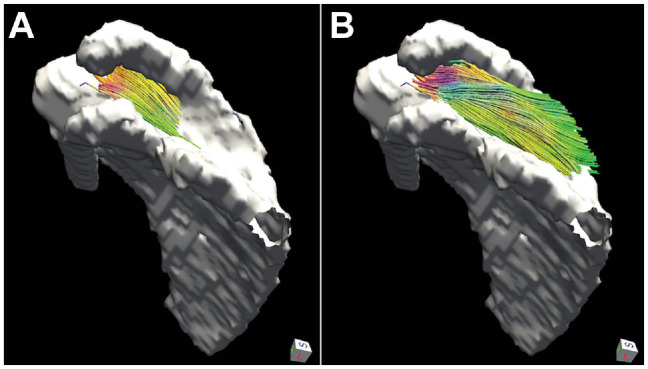
Diffusion tensor imaging tractography demonstrates the 3-dimensional relationship of muscle fibers in the (A) deep region of the supraspinatus versus the (B) superficial region. Color indicates directionality (blue, superior-inferior; red, left-right; green, anterior-posterior).

The deep fibers of the supraspinatus are visually depicted by DTI tractography in Figure 5. These fibers originate medial to and at the scapular notch and insert into the inferior margin of the main supraspinatus tendon. Reconstructions of muscle fiber length and orientation using tractography clearly show that these fibers are unique to the supraspinatus and distinct from the infraspinatus. Using tractography to roughly outline the deep fibers of the supraspinatus, this area of the muscle comprises an estimated 6% of the total supraspinatus muscle volume.

## Discussion

The main findings of this study demonstrated that the deep region of the supraspinatus was architecturally distinct from the anterior and posterior regions. The deep region had a significantly lower pennation angle. The fiber lengths were shorter—both raw and normalized. Most importantly, the deep region had fewer but longer sarcomeres. In contrast, the anterior and posterior regions were more similar. There were no differences in sarcomere length or sarcomere number between the anterior and posterior regions. Raw fiber length was significantly different, but it was similar once normalized. Consequently, we may speculate that the unique architecture of this deep “submuscle” region could imply differences in function compared with the rest of the supraspinatus.

Understanding the role of fewer, longer sarcomeres is very important. Previously, the optimal sarcomere length for any human muscle has been reported as 2.7 μm.^26^ The mean sarcomere length of the supraspinatus in the literature is 3.11 μm.^17^ The mean sarcomere lengths for the posterior and anterior regions found here (3.1 ± 0.2 μm and 3.2 ± 0.2 μm, respectively) are similar to the reported mean of 3.11 μm. This places the superficial supraspinatus muscle on the descending limb of the sarcomere length-tension curve.^9^ However, the deep region’s mean sarcomere length measured 3.4 ± 0.2 μm. These deep sarcomeres operate even further down the descending limb of the length-tension curve. In this segment of the curve, an increase in sarcomere length leads to less active force production, due to the decreasing interaction between actin and myosin myofilaments.^15,26^ Active tension is decreased, and conversely, passive tension is increased on this part of the curve. Greater resting passive tension and longer sarcomere lengths could imply a more significant position-sensing role of this deep submuscle region.

Sarcomere numbers are infrequently reported in other supraspinatus studies, especially compared with pennation angle or fiber length, yet it provides important insight into muscle function. Sarcomeres in series must contract to decrease muscle length and subsequently increase tendon excursion. A decrease in sarcomere number requires greater changes in sarcomere length to produce excursion. Therefore, having fewer sarcomeres leads to a higher sarcomere strain for a given change in muscle length or joint position.^2,4-7,28^ Again, we theorized that increased strain might mean that this area of the muscle is very sensitive to joint positions (ie, more strain on muscle spindles).

Similarly, shorter normalized fiber lengths in a muscle with 1 tendon and moment arm profile imply larger strains per unit joint angle. Both raw and normalized fiber lengths are decreased in the deep region. The supraspinatus muscle already has the shortest fibers among the rotator cuff muscles.^26^ Additional shortening of fibers leads to increased velocity and greater excursion imposed on the muscle by the joint.^3^ In contrast, the anterior and posterior region showed similar fiber lengths after normalization. The anterior region has previously been reported to have longer fiber bundle lengths, but these values were raw caliper measurements and not normalized.^18^

Rotator cuff tears have previously been shown to affect muscle architecture.^7,8,21,29^ It has been shown that muscle cross-sectional area and fiber size cross-sectional area decrease (ie, classic definitions of atrophy), but full-thickness tears also seem to adapt via serial sarcomere subtraction (fiber length shortening) to preserve sarcomere length.^7^ However, massive tears with retraction demonstrate both fewer and shorter sarcomeres.^7^ This alteration in muscle architecture may contribute to repair failure in massive tears, due to increased muscle tension and strain.^7^ In the present study, massive tears were not analyzed separately from other full-thickness tears due to limited sample size. Partial-thickness tears were associated with sarcomere shortening in the deep region, and full-thickness tears demonstrated higher pennation angles in the posterior region. These results should be interpreted cautiously, as there were only 3 partial-thickness tears and 9 full-thickness tears studied. The primary objective of this study was to delineate the regional differences within intact specimens. Future research could concentrate further on the effects of different-sized tears on the deep supraspinatus. Additionally, it should be investigated whether delaminated (articular-sided) tears of the supraspinatus may be explained by the presence of this deep muscle region and its different architectural or mechanical properties compared with the remainder of the supraspinatus.

### Noninvasive Assessment of Muscle Architecture

Muscle tractography is a powerful postprocessing tool that allows a user to approximate muscle fiber orientation (ie, pennation angle) and length in 3D from DTI-MRI data. This technique can be used to key architectural parameters of muscle noninvasively, supporting region-specific architectural analysis of muscle. Tractography was used in this study to visualize the deep supraspinatus fibers, which had a distinct origin, pennation angle, and length compared with the other fibers in the supraspinatus. Using tractography estimates, the deep region of the supraspinatus makes up approximately 6% of the total muscle volume. This technique can be applied in the future to quantify changes in supraspinatus architecture over time in response to rotator cuff tear and repair.

### Limitations

This study is not without limitations. While the sample size for the intact rotator cuff specimens (n = 12) was large enough to capture important differences between regions, the number for full (n = 9) and partial (n = 3) was likely insufficient to show smaller effect sizes for the impact of tear on these architecture measurements. Effect of tear was a secondary outcome of the present study and has been studied extensively by other authors.^7,8,21,29^ Another limitation inherent in cadaveric studies is that certain parameters are fixed (eg, joint angle) and that active motion cannot be captured. Fiber lengths were normalized to account for joint angle-dependent fiber length variation, at least. Finally, these measurements provide insight into architecture only and should not be extrapolated directly to conclusions about function. Further investigation is needed to characterize the functional and pathophysiological implications of this architecturally distinct region. Other points of interest include the biomechanical differences between the deep and superficial regions, the fiber type composition of the deep region, and how the architectural measurements change in vivo.

## Conclusion

The deep supraspinatus was found to have significantly lower pennation angles, shorter fiber lengths, and fewer but longer sarcomeres compared with other subregions. These structural differences suggest a functionally unique submuscle within the supraspinatus. The architectural changes in this region imply weaker active force production and higher passive resting tension in the anatomic position. Follow-up studies could examine the density or sensitivity of muscle spindles in this region, or compare electromyography of the deep versus superficial tissue, to better characterize this region’s role. If there is a difference in concentration of neural elements for proprioception and position sense (golgi organs, muscle spindles, etc), this would support the concept of a region-specific function of the deep fibers. Furthermore, differences in strain could be compared after muscle atrophy and/or tendon retraction.

## References

[1] Berry DB Rodriguez-Soto AE Englund EK , et al. Multiparametric MRI characterization of level dependent differences in lumbar muscle size, quality, and microstructure. JOR Spine. 2020;3(2):e1079. 10.1002/jsp2.1079 PMC7323468 32613159

[2] Boakes JL Foran J Ward SR Lieber RL. Muscle adaptation by serial sarcomere addition 1 year after femoral lengthening. Clin Orthop Relat Res. 2007;456:250-253. 17065842 10.1097/01.blo.0000246563.58091.af

[3] Burkholder TJ Fingado B Baron S Lieber RL. Relationship between muscle fiber types and sizes and muscle architectural properties in the mouse hindlimb. J Morphol. 1994;221(2):177-190. 7932768 10.1002/jmor.1052210207

[4] Burkholder TJ Lieber RL. Sarcomere length operating range of vertebrate muscles during movement. J Exp Biol. 2001;204(pt 9):1529-1536. 11296141 10.1242/jeb.204.9.1529

[5] Burkholder TJ Lieber RL. Sarcomere number adaptation after retinaculum transection in adult mice. J Exp Biol. 1998;201(pt 3):309-316. 9503642

[6] Felder A Ward SR Lieber RL. Sarcomere length measurement permits high resolution normalization of muscle fiber length in architectural studies. J Exp Biol. 2005;208(pt 17):3275-3279. 16109889 10.1242/jeb.01763

[7] Gibbons MC Sato EJ Bachasson D , et al. Muscle architectural changes after massive human rotator cuff tear. J Orthop Res. 2016;34(12):2089-2095. 27061583 10.1002/jor.23256 PMC5423410

[8] Hayashi I Enokida M Nagira K , et al. Change in the pennation angle of the supraspinatus muscle after rotator cuff tear repair. J Shoulder Elbow Surg. 2019;28(5):888-892. 30799200 10.1016/j.jse.2018.10.021

[9] Hill AV. The dynamic constants of human muscle. Proc R Soc Lond B. 1940;128(852):263-274.

[10] Kim S Bleakney R Boynton E , et al. Investigation of the static and dynamic musculotendinous architecture of supraspinatus. Clin Anat. 2010;23(1):48-55. 19941361 10.1002/ca.20896

[11] Kim SY Boynton EL Ravichandiran K , et al. Three-dimensional study of the musculotendinous architecture of supraspinatus and its functional correlations. Clin Anat. 2007;20(6):648-655. 17352416 10.1002/ca.20469

[12] Kim SY Sachdeva R Li Z Lee D Rosser BW. Change in the pathologic supraspinatus: a three-dimensional model of fiber bundle architecture within anterior and posterior regions. Biomed Res Int. 2015;2015:564825. 26413533 10.1155/2015/564825 PMC4564630

[13] Lieber RL Fazeli BM Botte MJ. Architecture of selected wrist flexor and extensor muscles. J Hand Surg Am. 1990;15(2):244-250. 2324452 10.1016/0363-5023(90)90103-x

[14] Lieber RL Friden J. Functional and clinical significance of skeletal muscle architecture. Muscle Nerve. 2000;23(11):1647-1666. 11054744 10.1002/1097-4598(200011)23:11<1647::aid-mus1>3.0.co;2-m

[15] Lieber RL Ward SR. Skeletal muscle design to meet functional demands. Philos Trans R Soc Lond B Biol Sci. 2011;366(1570):1466-1476. 21502118 10.1098/rstb.2010.0316 PMC3130443

[16] Lieber RL Yeh Y Baskin RJ. Sarcomere length determination using laser diffraction: effect of beam and fiber diameter. Biophys J. 1984;45(5):1007. 10.1016/S0006-3495(84)84246-0 PMC1434983 6610443

[17] Mathewson MA Kwan A Eng CM Lieber RL Ward SR. Comparison of rotator cuff muscle architecture between humans and other selected vertebrate species. J Exp Biol. 2014;217(pt 2):261-273. 24072803 10.1242/jeb.083923 PMC3898624

[18] Roh MS Wang VM April EW , et al. Anterior and posterior musculotendinous anatomy of the supraspinatus. J Shoulder Elbow Surg. 2000;9(5):436-440. 11075329 10.1067/mse.2000.108387

[19] Sacks RD Roy RR. Architecture of the hindlimb muscles of cats: functional significance. J Morphol. 1982;173(2):185-195. 7120421 10.1002/jmor.1051730206

[20] Thompson SM Reilly P Emery RJ Bull AM. An anatomical description of the pennation angles and central tendon angle of the supraspinatus both in its normal configuration and with full thickness tears. J Shoulder Elbow Surg. 2011;20(6):899-903. 21454103 10.1016/j.jse.2011.01.005

[21] Tomioka T Minagawa H Kijima H , et al. Sarcomere length of torn rotator cuff muscle. J Shoulder Elbow Surg. 2009;18(6):955-959. 19515583 10.1016/j.jse.2009.03.009

[22] Vahlensieck M Haack Ka Schmidt HM. Two portions of the supraspinatus muscle: a new finding about the muscles macroscopy by dissection and magnetic resonance imaging. Surg Radiol Anat. 1994;16(1):101-104. 8047956 10.1007/BF01627931

[23] Vahlensieck M Pollack M Lang P Grampp S Genant HK. Two segments of the supraspinous muscle: cause of high signal intensity at MR imaging? Radiology. 1993;186(2):449-454. 8421749 10.1148/radiology.186.2.8421749

[24] Wang R Benner T Sorensen AG Wedeen V. Diffusion toolkit: a software package for diffusion imaging data processing and tractography. Proc Int Soc Magn Reson Med. 2007;15:3720.

[25] Ward AD Hamarneh G Ashry R Schweitzer ME. 3D shape analysis of the supraspinatus muscle: a clinical study of the relationship between shape and pathology. Acad Radiol. 2007;14(10):1229-1241. 17889340 10.1016/j.acra.2007.06.014

[26] Ward SR Hentzen ER Smallwood LH , et al. Rotator cuff muscle architecture: implications for glenohumeral stability. Clin Orthop Relat Res. 2006;448:157-163. 16826111 10.1097/01.blo.0000194680.94882.d3

[27] Ward SR Sarver JJ Eng CM , et al. Plasticity of muscle architecture after supraspinatus tears. J Orthop Sports Phys Ther. 2010;40(11):729-735. 20710096 10.2519/jospt.2010.3279 PMC4321894

[28] Winters TM Takahashi M Lieber RL Ward SR. Whole muscle length-tension relationships are accurately modeled as scaled sarcomeres in rabbit hindlimb muscles. J Biomech. 2011;44(1):109-115. 20889156 10.1016/j.jbiomech.2010.08.033 PMC3003754

[29] Zuo J Sano H Itoi E. Changes in pennation angle in rotator cuff muscles with torn tendons. J Orthop Sci. 2012;17(1):58-63. 22094606 10.1007/s00776-011-0176-6

